# Temperature-induced formation of Pd nanoparticles in heterogeneous nanobiohybrids: application in C–H activation catalysis†

**DOI:** 10.1039/d2na00742h

**Published:** 2022-12-20

**Authors:** Noelia Losada-Garcia, A. Sofia Santos, M. Manuel B. Marques, Jose M. Palomo

**Affiliations:** a Instituto de Catálisis y Petroleoquímica (ICP), CSIC C/Marie Curie 2 28049 Madrid Spain josempalomo@icp.csic.es; b LAQV@REQUIMTE, Department of Chemistry, NOVA School of Science and Techonology. Universidade Nova de Lisboa 2829-516 Caparica Portugal

## Abstract

The effect of the temperature in the synthesis of Pd nanoparticles in the metal-enzyme biohybrids is evaluated. The effect on the formation, size, and morphology of nanoparticles was evaluated using *C. antarctica* B lipase as the protein scaffold. XRD analyses confirmed the formation of crystalline Pd(0) as the metal species in all cases. TEM analyses revealed spherical crystalline nanoparticles with average diameter size from 2 nm at 4 °C synthesis to 10 nm obtained at 50 °C synthesis. The thermal phenomenon was also critical in the final hybrid formation using more complex enzymes, where the relation of the protein structure and temperature and the influence of the latter has been demonstrated to be critical in the reducing efficiency of the enzyme in the final Pd nanoparticle formation, in the metal species, or even in the final size of the nanoparticles. Different Pd biohybrids were evaluated as catalysts in the C–H activation of protected l-tryptophan under mild conditions. Pd@CALB4 showed the best results, with >99% conversion for *C*-2 arylation in methanol at room temperature with a TOF value of 64 min^−1^, being 2 or 4 times higher than that of the other synthesized hybrids. This catalyst showed a very high stability and recyclability, maintaining >95% activity after three cycles of use.

## Abstract 2

Effect of temperature on the synthesis of enzyme–PdNP hybrids for highly efficient C–H activation catalysts.

## Graphical abstract

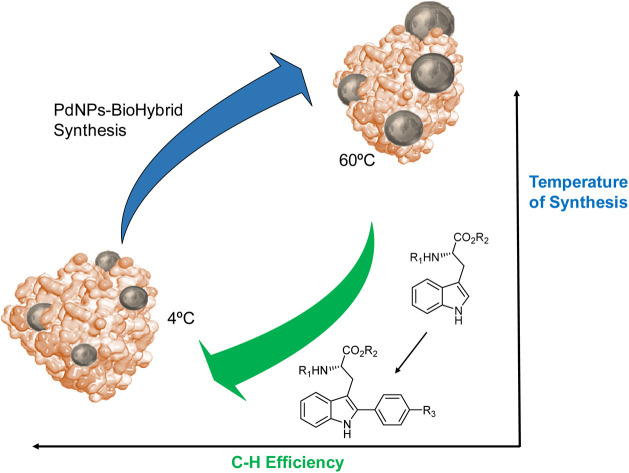

## Introduction

Palladium represents probably the most used transition metal elements in catalysis with thousands of applications in many different types of reactions.^1–8^ From the different compounds, Pd nanoparticles (PdNPs) have gained a high leadership in catalytic performance in recent years,^9–16^ due in part to the intrinsic properties of nanoparticles compared to those of bulk materials.

From the different synthetic protocols to obtain metal nanoparticles, the use of isolated biological entities such as enzymes have embodied an excellent pioneer strategy.^17–21^ This methodology allows the synthesis of metal nanoparticles in heterogeneous form under mild conditions, especially in aqueous media and air.

The enzyme has a key role in the creation of the metal nanoparticles embedded on the protein matrix. First, simple metal ions in solution are coordinated with specific amino acid residues on the protein structure (*i.e.*, hydrophobic or negative charged side chains). This binding process caused a precipitation of crosslinked metal ions–protein complexes. The second role of the protein is based on the *in situ* reduction of Pd^2+^ to Pd(0). This process goes through particular amino acid residues near the binding sites from the same peptide chain or nearly three-dimensional located, showing a strong reducing ability (*i.e.*, amino acids presenting hydrophobic or hydroxyl side chains). After reduction, metal particles are formed and it is the last step is the growth of the nanoparticle. This step is controlled by the protein network previously formed and is an important aspect in the final size of the nanoparticles.

Therefore, changes in the structural conformation of the protein would have an important effect on the final properties of the nanobiohybrid, especially in the formation of metal nanoparticles on them, by morphology, size, or even metal species.

Recently, the alteration of experimental conditions such as pH change (affecting the isoelectric point of the protein) or the presence of additives in the synthetic process (detergent, ionic polymers) have demonstrated changes in the properties of the metal nanoparticles synthesized.^22–30^

In particular, temperature (*T*) is one of the most critical parameters in terms of enzyme stability. Most of enzymes showed lower stability at relative high temperature, even at moderate temperature. The alteration of temperature caused important secondary and especially tertiary structural changes in the protein. The increase in the protein flexibility at higher temperature or the increase in the rigidity of the structure at lower *T* could be translated in the final size of the metal nanoparticles, considering that the protein network has an important role in this.^31^

The growth of the NPs as a function of the enzyme structure is another important parameter. Enzymes present very different amino acid sequences and different location of the key amino acids in three-dimensional form. This means that one protein could have many binding sites but not enough neighboring reducing groups, which can be translated to the final lower amount of the nanoparticles.^29^ In this term, more complex examples could be the use of multimeric enzymes, where the whole protein is a dimeric or tetrameric structure.

Thus, the combination of experimental conditions affecting the enzyme and structural characteristic of enzymes *per se* in the induced formation of NPs in the synthesis of Pd-enzyme nanobiohybrids has been evaluated in this work, in terms of the speed of the synthesis, final morphology, size of the nanoparticles, and final effect on the Pd species or Pd deposition on the protein network.

The different novel synthesized Pd nanobiohybrids were tested as catalysts in the site-selective C–C bonding modification of indoles, which are privileged scaffolds with many applications in the field of medicinal chemistry.^32–34^

**Scheme 1:**
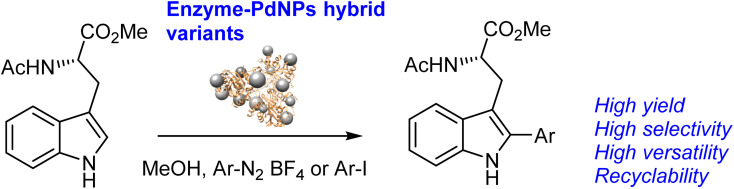
Selective C–H activation of tryptophan derivatives catalyzed by Pd nanobiohybrids.

The functionalization of tryptophan in *C*-2 by C–H activation was performed by Pd-catalysis for drugs total synthesis.^35^ Different organometallics, palladium salts, or immobilized systems have been applied in this reaction; however, in all cases, longer time reaction or high *T* has been necessary (Scheme S1†).^26,36–38^ Herein, we successfully demonstrated our Pd strategy for simple, efficient, and rapid C–H activation (Scheme 1).

## Experimental section

### Chemicals

Lipase B from *Candida antarctica* (Lipozyme® CALB), *Thermomyces lanuginosus* lipase (Lipozyme® TL 100L, TLL), catalase from *Aspergillus niger* (Catazyme® 25L, CAT), and β-galactosidase from *Kluyveromyces* lactis (Lactozyme® pure 6500L, LAC) solutions were from Novozymes (Denmark). 4-Methoxybenzenediazonium tetrafluoroborate (MBDFB), iodobenzene, palladium acetate, sodium tetrachloropalladate (Na_2_[PdCl_4_]), and CuSO_4_·5H_2_O were from Sigma-Aldrich (now Merk). *N*-Acetyl-l-tryptophan methyl ester (TrpOMe) was from Alfa Aesar. Methanol and acetonitrile of HPLC grade were purchased from Scharlab (Scharlab, S.L., Barcelona, Spain). Cu-FOS2 and Cu-NR hybrids were synthesized following the previously reported strategy.^18^

### Structural characterization

Inductively coupled plasma-optical emission spectrometry (ICP-OES) was performed on an OPTIMA 2100 DV instrument (PerkinElmer, Waltham, MA, USA). X-Ray diffraction (XRD) patterns were obtained using a Texture Analysis D8 Advance Diffractometer (Bruker, Billerica, MA, USA) with Cu Kα radiation. Transmission electron microscopy (TEM) and high-resolution TEM microscopy (HRTEM) images were obtained on a 2100F microscope (JEOL, Tokyo, Japan). Scanning electron microscopy (SEM) imaging was performed on a TM-1000 microscope (Hitachi, Tokyo, Japan). To recover the biohybrids, a Biocen 22 R (Orto-Alresa, Ajalvir, Spain) refrigerated centrifuge was used. X-ray photoelectron spectroscopy (XPS) spectra were determined through a SPECS GmbH electronic spectroscopy system with a UHV system (pressure approx. 10–10 mbar), with a PHOIBOS 150 9MCD energy analyzer, monochromatic X-ray sources. The analysis of the same was carried out using the CasaXPS program.

### Analytical characterization

HPLC analysis was performed in a JASCO HPLC equipment, a HPLC pump PU-4180 coupled with a UV-4075 UV-Vis detector. Samples of the reaction mixture (50 μL) were centrifuged and then 25 μL was diluted in 1 mL 50 : 50 ACN : water before the injection. The analysis conditions were achieved with a Kromasil-C8 (150 × 4.6 mm and 5 μm *ø*) at a flow of 1.0 mL min^−1^, *λ*: 270 nm, and 50% (v/v) ACN in MilliQ water as the mobile phase. The configuration was determined by HPLC using the sample standards.

### Synthesis of Pd@CALB biohybrids

Pd(OAc)_2_ (20 mg) was dissolved in 4 mL MeOH. At the same time, 720 μL Lipase B from *Candida antarctica* solution (CALB; 10 mg mL^−1^) was dissolved in 16 mL distilled water. Palladium solution was added to the enzyme solution. The presence of the co-solvent (20% v/v) was necessary for the complete dissolution of the Pd salt in aqueous medium. The mixture was kept under gentle stirring for 72 h at 4 °C, 24 h at 25 °C, 50 °C, or 60 °C. After that, the final suspension was separated by centrifugation (8000 rpm, 10 min) and the resulting solid was washed with the same solution (water with 20% v/v MeOH) and, finally, with distilled water (twice). After the last washing, 2 mL distilled water was added to the solid, the suspension was frozen in liquid nitrogen, and lyophilized, obtaining a dry solid called Pd@CALB4, Pd@CALB25, Pd@CALB50, and Pd@CALB60 with a solid yield of 23 mg, 24.8 mg, 15.7 mg, and 23 mg, respectively.

A similar synthetic protocol was followed for biohybrid preparation with other enzymes, where the amount of the enzyme added in each case was 242 μL of TLL (29.8 mg mL^−1^) and 24 h incubation time at 25 °C for Pd@TLL25 (14.2 mg); 288 μL of CAT (25 mg mL^−1^) and 24 h at 50 °C for Pd@CAT50 (0.6 mg); 147 μL of LAC (49 mg mL^−1^) and 12 h at 70 °C for Pd@LAC70 (9.4 mg).

### C–H activation of *N*-acetyl-l-tryptophan methyl ester (1) catalyzed by Pd@enzyme hybrids

0.049 mmol (10 mg) *N*-acetyl-l-tryptophan methyl ester (1) and 0.049 mmol (11 mg) MBDFB (2) or 0.1029 mmol (9 μL) of iodobenzene (4) were added to a glass flask containing 1 mL MeOH or 1 mL 50 : 50 MeOH : water. The solution was left under magnetic stirring until homogenization. Then, 1 mg Pd@enzyme biohybrids alone or in combination with Cu hybrids (1 mg or 10 mg) was added. The mixture was kept at room temperature or 40 °C. The outgoing reaction mixture was monitored by the HPLC analysis of the reaction mixture's samples withdrawn at different times.

### Hot filtration catalyst experiment

After 1 h reaction in the following conditions, 100% methanol and 21 °C, Pd@CALB4 catalyst was centrifuged and separated from the reaction mixture. A small amount of water was added to the solid and this was incubated at 50 °C for 30 min. Then, the catalyst was centrifuged and the solid was recovered and separated from the supernatant. ICP-OES analysis of the supernatant demonstrated the absence of any traces of palladium.

## Results and discussion

### Synthesis of enzyme–Pd nanoparticles hybrids at different *T*

The nanobiohybrids were synthesized using the commercial liquid CAL-B. The protocol involved the use of palladium acetate as the salt and distilled water containing 20% (v/v) of methanol as the solvent. Synthesis was performed at 4 °C, 25 °C, 50 °C, and 60 °C. In all cases, a heterogeneous material was obtained after a corresponding incubation time, with all the enzyme added disappeared from the solution (Table S1†). Sodium tetrachloropalladate was also used in fully aqueous media as the palladium source, but after 24 h incubation, almost no heterogeneous material was formed, and about 90% of the offered enzyme still remained on the supernatant (measure by Bradford method) (data not shown).

**Fig. 1:**
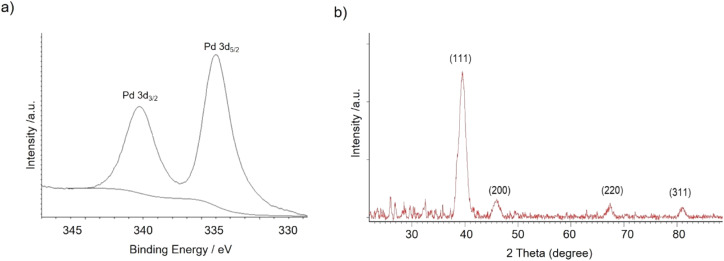
(a) XPS spectrum of Pd 3d. (b) XRD spectrum of Pd(0) in nanobiohybrids.

X-ray diffraction (XRD) and X-ray photoelectron spectroscopy (XPS) analysis demonstrated that Pd(0) was the only metal species in the nanobiohybrid in all the cases (Fig. 1).

**Fig. 2:**
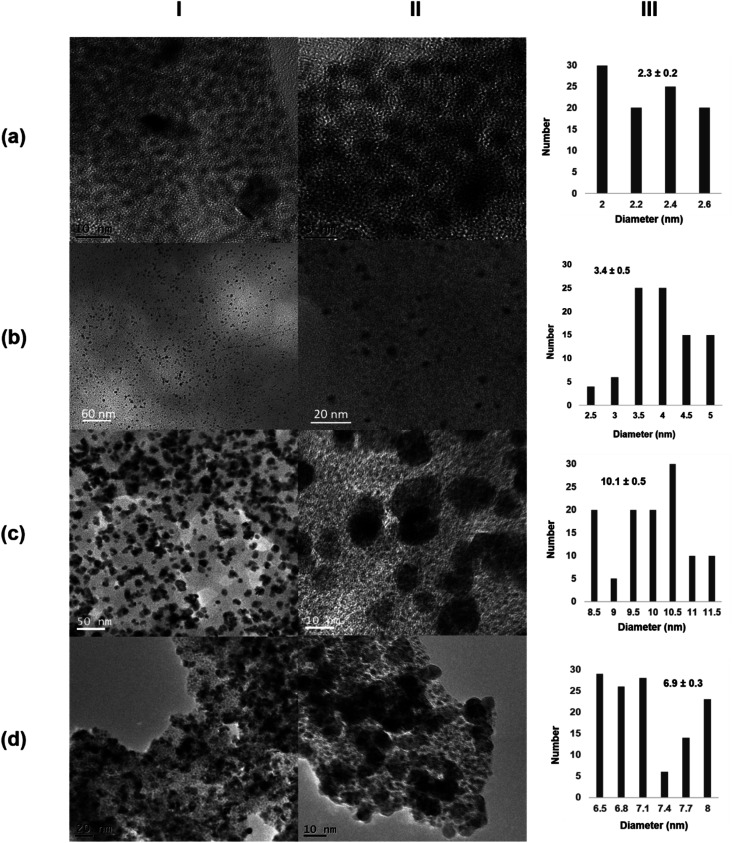
Characterization of different Pd@CALB biohybrids. (a) Pd@CALB4; (b) Pd@CALB25; (c) Pd@CALB50; (d) Pd@CALB60. (I) TEM images; (II) HR-TEM images; (III) nanoparticles' size distribution.

The morphology and the distribution of such metallic nanoparticles embedded in the enzymatic net were investigated by transmission electron microscopy (TEM) and high-resolution TEM microscopy (HRTEM) for all the four synthesized biohybrids (Pd@CALB4, Pd@CALB25, Pd@CALB50, Pd@CALB60) (Fig. 2 and S1–S3†). TEM analysis revealed the formation of Pd nanoparticles (PdNPs) without using any reducing agent during the synthesis. Fig. 2 shows that the morphology, diameter size, and distribution of the formed palladium nanoparticles were quite different depending on the temperature used in the synthesis (Fig. 2 and Table S1†). The synthesis at 4 °C (Pd@CALB4) produced a biohybrid containing the smallest PdNPs, showing an average diameter size of 2 nm (Fig. 2a).

The increase in the temperature from 4 to 25 °C during the synthesis caused a clear increase in the nanoparticle average size in the Pd@CALB25 hybrid, producing mainly spherical nanoparticles with a diameter average size of about 3–4 nm, with a very minor fraction of nanoparticles of up to 7 nm (Fig. 2b). This effect could be explained considering the flexibility of the protein structure, which increases at 25 °C.^27^

The reducing capacity of the enzyme was also affected in terms of the deposited Pd yield, which increased from 14% to 31% when *T* increased from 4 to 25 °C (Tables S1†).

Higher temperatures are related to an enhancement in the structural flexibility of the protein but also affect the folding–unfolding equilibrium of the protein. In the case of CALB, the enzyme is still stable at 50 °C, whereas the structure starts unfolding at 60 °C.^39^

The Pd-hybrid synthesis at 50 °C (Pd@CALB50) showed a faster formation of the palladium nanoparticles, although the final Pd deposited yield was lower than that obtained at 25 °C (18%, Tables S1†). This could be explained considering the aromatic groups, which are quite important for the thermostability of the proteins, and weaker hydrophobic interactions can be observed at lower or higher T. In this case, the increase from 25 to 50 °C could cause these weaker interactions (also in key amino acid residues), therefore decreasing the Pd *in situ* reducing efficiency of the CALB. In the nanoparticle size, an increase in size from about 3 to 10 nm was observed (Fig. 2c), also caused by a higher protein flexibility; therefore, the nanoparticle growth control was reduced.

The synthesis of the hybrid was also attempted at 60 °C, and surprisingly, the Pd@CALB60 hybrid contained Pd nanoparticles with an average diameter size of 6.9 nm, smaller than that observed at 50 °C (Fig. 2d). Also, the Pd deposited increased (25%, Table S1†), which shows that the reducing protein effect was also higher than at 50 °C. This could be explained considering the presence of partial unfolded protein, which could reduce the metal ions faster and acts as an “additive” in the protein–Pd conjugate in the control of particle growth.

**Fig. 3:**
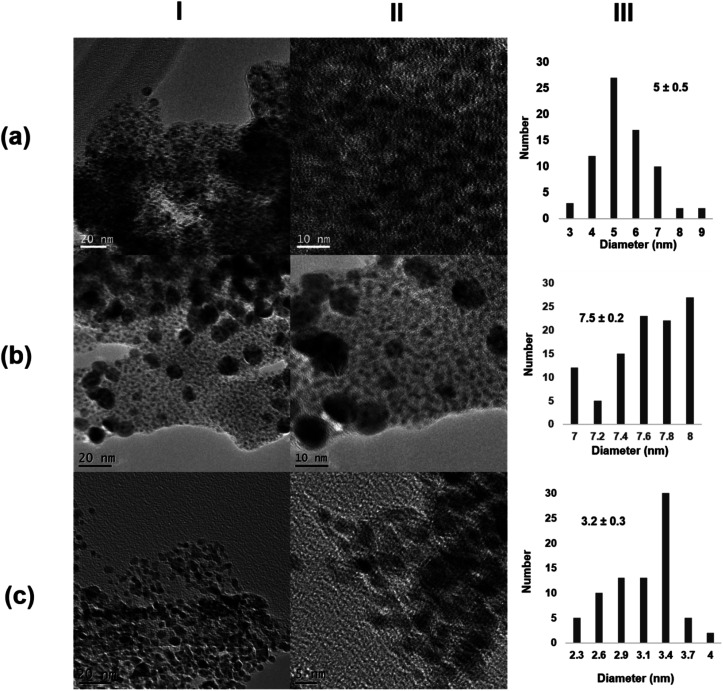
Characterization of the different Pd@enzymes biohybrids. (a) Pd@TLL25; (b) Pd@CAT50; (c) Pd@LAC70. (I) TEM images; (II) HR-TEM images; (III) nanoparticles size distribution.

Next, the role of the protein scaffold was evaluated. First, an enzyme with similar molecular weight as that of monomeric CALB (33 kDa), *Thermomyces lanuginosus* lipase (TLL), but with clear structural difference (*e.g.*, much more carboxylic groups on the surface) and a high tendency to form dimer structures^40^ was used at 25 °C. In this case, XRD analysis indicated the presence of Pd/PdO species in the Pd@TLL25 hybrid (Fig. S4†). This extra-oxidation of Pd could be due to the five histidine existing in the protein sequence, which could also serve as the coordinating groups for Pd (Fig. S5†). The TEM images showed the formation of spherical nanoparticles with an average diameter size of 5 nm (Fig. 3a and S4†), larger than using CALB as the scaffold. This could be due to the larger size of the protein, which mainly exists in the dimeric form in solution. The enzymatic reducing efficiency was, however, slight lower than that of CALB (24% of Pd deposited yield) (Table S1†). The second approach was the use a multimeric enzyme (240 kDa), catalase from *Aspergillus niger* (CAT). The synthesis of the Pd hybrid was attempted at 4, 25, and 35 °C, but in these cases, no solid aggregate was observed. Then, the temperature was increased at 50 °C, at which the enzyme starts to be inactivated.^41^ After a longer incubation time, a slight solid Pd@CAT50 was generated; however, the reduction efficiency of this enzyme was very low, with only 1% of deposited Pd yield (Table S1†). This could be explained by evaluating the three-dimensional structure of the enzyme, which was obtained by the artificial intelligence program alpha-fold because no crystal structure was still available (Fig. S6†). The main carboxylic groups in the multimeric structure are quite near to positive residues, generating salt bridges at the synthetic conditions; therefore, they are not accessible to coordinate the Pd atoms (Fig. S7a†). Also, reducing amino acid residues (Cys, Ser, or Tyr) near to this group are not able to carry out their function (Fig. S7b†). The increase in *T* causes an increase in the protein flexibility, which could make some of the groups inaccessible, but in low amount; therefore, almost no hybrid was formed. XRD analysis showed that Pd(0) was the unique species, while the TEM images showed the formation of nanoparticles with about 7.5 nm size (Fig. 3b and S8†).

In the third case, a much larger multimeric protein was used as the protein scaffold, namely, β-galactosidase from *Kluyveromyces lactis* (LAC) with a molecular weight of 475 kDa.^42^ Pd-hybrid synthesis was performed at different temperatures, similar to that with CAT, but even at 50 °C, no solid was obtained. In this case, a similar explanation to that using CAT can be given by analyzing the three-dimensional structure of LAC (Fig. S9 and S10†). Thus, an increase in the temperature to 70 °C was done. This enzyme is, however, not very stable at this temperature, where the unfolding process occurs. At these conditions, a similar reducing effect as that of with 30% Pd deposition yield (Table S1†) and clear formation of a solid aggregate was observed. The TEM images showed very small nanoparticle sizes of about 3 nm, although a large protein was used (Fig. 3c and S11†). This could be explained considering the potential inactivation of the protein at high temperature where very specific groups were available for coalescence and growth, thus controlling this latter by the quaternary structure.

### Catalytic efficiency of the different Pd-hybrids in the C–H activation process

First, the Pd biohybrids were used as catalysts at different conditions in the selective C–H activation of *N*-acetyl-l-tryptophan methyl ester (1) with 4-methoxybenzenediazonium tetrafluoroborate (2) to obtain C–H product at *C*-2 position of the indole moiety in the Trp residue (3) (Table 1).

**Table 1:** C–H activation of *N*-acetyl-l-tryptophan methyl ester (1) catalyzed by different Pd@enzyme hybrids^a^

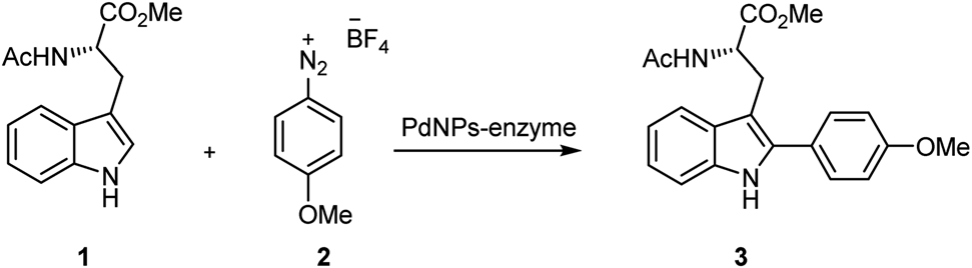						
Entry	Catalyst	Solvent	Time (h)	Conversion of 3^e^ (%)	Selectivity^f^ (%)	TOF value^g^ (h^−1^)
1	Pd@CALB4	MeOH	1	98	>99	64.10
2	Pd@CALB25	MeOH	1	95	>99	38.54
3	Pd@CALB50	MeOH	1	89	>99	36.50
4	Pd@CALB60	MeOH	1	97	>99	44.09
5	Pd@TLL25	MeOH	3	94	>99	9.66
6	Pd@CAT50	MeOH	96	61	>99	0.05
7	Pd@LAC70	MeOH	96	76	>99	0.03
8	Pd@CALB4	MeOH^b^	1	84	>99	19.47
9	Pd@CALB4	MeOH : H_2_O^c^	4	90	>99	13.56
10	Pd@CALB4	MeOH : H_2_O^d^	4	72	>99	6.26
11	Pd@CALB25	MeOH : H_2_O^c^	4	70	>99	11.79
12	Pd@CALB60	MeOH : H_2_O^c^	4	88	>99	11.38

a Conditions: 1 (0.049 mmol), 2 (0.049 mmol), solvent (1 mL), catalyst (1 mg), r.t (*ca.* 21 °C).

b 40 °C.

c (50 : 50 v/v).

d (80 : 20 v/v).

e Conversion of product 3 was quantified by HPLC.

f Selectivity as unique product, determined by HPLC.

g TOF value was calculated considering the conversion between 20 and 50% in each case.

The CALB-Pd hybrids exhibited more than 95% conversion of 3 with excellent selectivity in 1 h when synthesis was performed in methanol at r.t (Table 1, entries 1, 2 and 4), except Pd@CALB50, which showed 89% (Table 1, entry 3). In terms of efficiency, Pd@CALB4 was the best catalyst, with the highest TOF value, 64 h^−1^ (Table 1, entry 1), almost two times higher than that from other CALB hybrids. This result shows the important effect of the temperature of the synthesis related to the nanoparticle size in the final efficiency of the catalyst, presenting a greater active catalytic surface.

Also, another factor tested was the enzyme scaffold. Pd@TLL25 yield 94% of 3 in 3 h, showing lower effectiveness, with a TOF value 3-fold lower compared with that of Pd@CALB25. This could be explained considering the presence of PdO as the species and also the composition of larger nanoparticles. Pd@CAT50 and Pd@LAC70 showed much lower catalytic efficiency, making 96 h reaction necessary to produce about 60–70% conversion (Table 1, entries 6 and 7). This could be due to the effect of the protein's environment, which affects the protein stability in the presence of pure solvent.^41,42^

To evaluate the experimental conditions in the synthesis, the reaction was catalyzed by Pd@CALB4 at 40 °C or in the presence of water as the additive. In the former, conversion was slightly lower (84% in 1 h), but the TOF value decreased 3 times (Table 1, entry 8). In the latter, the aqueous medium did not improve the results (Table 1, entries 9 and 10). The negative effect on the conversion in the presence of water was also observed using Pd@CALB25 and Pd@CALB60. This could be explained considering an alteration in the reaction kinetically, as has been recently observed using Pd catalyst in dehydrogenation reactions between methanol and water.^43^

Finally, to demonstrate the heterogeneity of the catalyst, the hot filtration experiment was performed as described in the experimental section for Pd@CALB4 in these reaction conditions, with the solid material being recovered in all.

To improve the results, a combination with another metal, such as copper, well-known in the activation of Pd catalysis in several C–C bond formation reactions,^44,45^ was evaluated. In these terms, the reaction was performed with Pd@CALB4 using two different Cu biohybrids as the additives synthesized previously, Cu-NR (containing Cu(ii) nanoparticles), and Cu-FOS2 (containing Cu(i), Cu_2_O nanoparticles) (Fig. S12†).^18^ First, in both cases, Cu-catalysts were evaluated in the presence of 2 being stable in methanol but not in the methanol/water mixture (data not shown). Unfortunately, 1 equiv. Cu(i) caused a very low conversion of C–H activation and high amount of undesired 4-methoxybenzene. However, using Cu(ii) catalysts, a unique product 3 was obtained in 24 h. The increase in 10 equiv. of Cu(ii) catalyst caused a low specificity and C–H conversion (Table 2).

**Table 2:** C–H activation of *N*-acetyl-l-tryptophan methyl ester (1) catalyzed by Pd@CALB4 in the presence of Cu hybrid catalysts^a^

Entry	Additive	Conversion of 3^c^ (%)	Selectivity (%)
1	Cu-FOS2	43(59)^d^	>99
2	Cu-NR	94	>99
3	Cu-NR^b^	33(22)	>99

a Conditions: 1 (0.049 mmol), 2 (0.049 mmol), methanol (1 mL), reaction time (24 h), Pd@CALB4 (1 mg), additive (1 equiv.), r.t (*ca.* 21 °C).

b 10 equiv.

c Conversion of the product 3 was quantified by HPLC.

d Conversion of undesired 3-methoxybenzene.

This may be due to the accessibility of odd-numbered electron states in copper, which implies that copper may participate in single-electron redox transfer processes; therefore, an alternative free radical mechanism must be considered. It has been reported that these mechanisms prevail when arenediazonium salts are used as electrophilic reagents in Cu(i)-assisted nucleophilic substitution but cannot be ruled out with other leaving groups.^46^

**Fig. 4:**
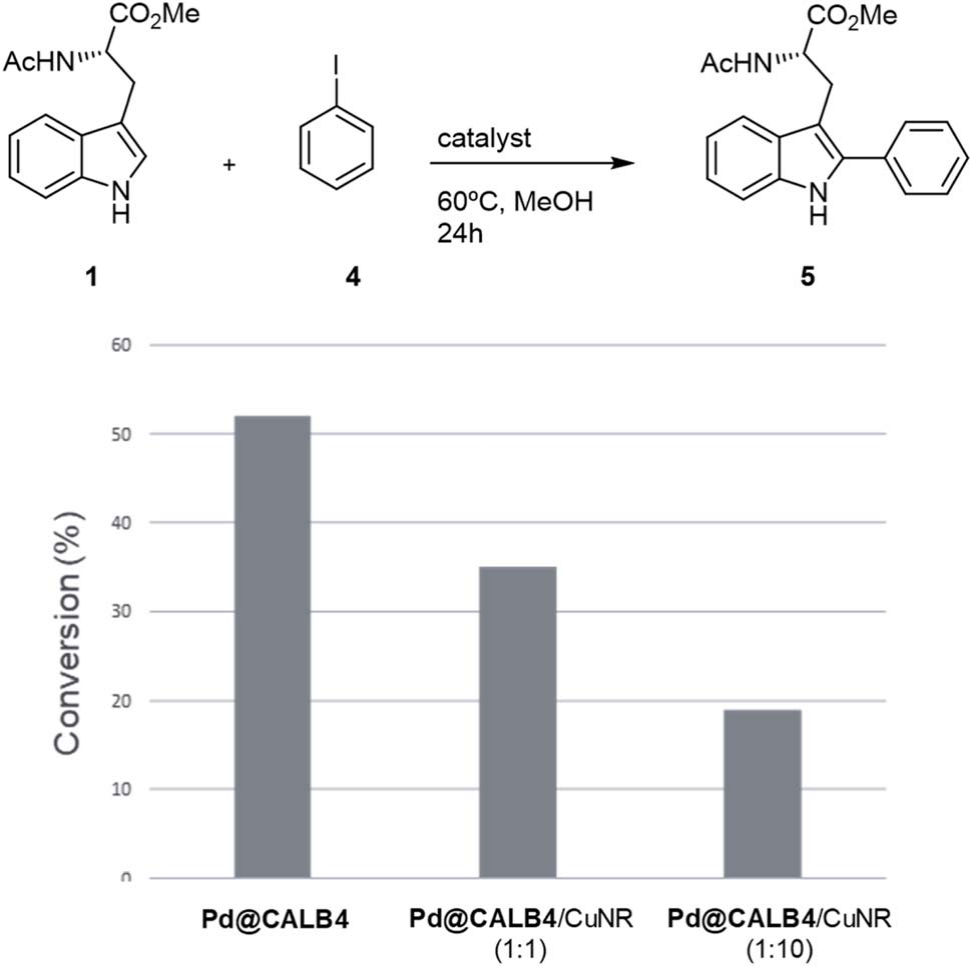
Application of Pd@CALB4 and Cu biohybrid for the C–H activation of 1 using iodobenzene (4). Reaction Conditions: 1 (0.046 mmol), 4 (0.1029 mmol), solvent (1 mL), 24 h at r.t (*ca.* 21 °C). Conversion of product 5 was quantified by HPLC.

To test the versatility of the methodology, Pd@CALB4 was evaluated in the C–H activation of 1 using iodobenzene (4) at 60 °C in methanol without additional base (Fig. 4). A conversion of 52% was obtained after 24 h incubation, with a TOF value of 0.75 h^−1^. In addition, the combination with Cu-NR catalyst was performed. No activity was observed using only Cu hybrid. However, as previously, the final conversion achieved by Pd catalysts was also less (Fig. 4). In addition, higher amount of Cu catalysts decreased the final efficiency of the Pd hybrids in C–H activation. These results demonstrate that Cu could affect the oxidative catalytic mechanism of Pd in C–C bond formation.

### Reusability of the catalyst

**Fig. 5:**
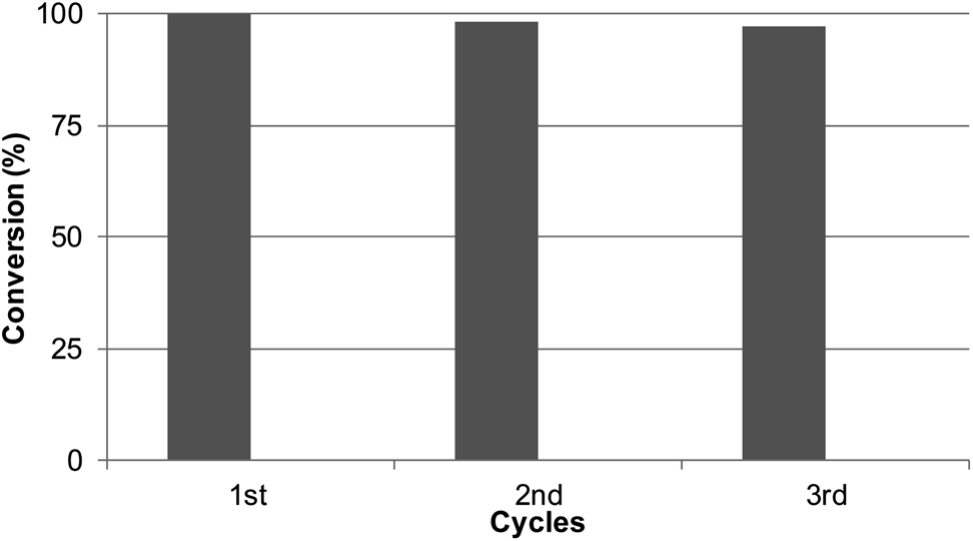
Reusability experiments of Pd@CALB4 in the C–H activation reaction of 1 to 3.

Considering the results obtained, the robustness of the catalysts Pd@CALB4 was tested by recycling experiments. The catalyst was used at least three times in the C–H reaction at r.t in methanol, and the conversion and selectivity were evaluated at each step. As shown in Fig. 5, the Pd-nanocatalyst retained the initial catalytic activity and selectivity, producing the *C*-2 product 3 exclusively.

## Conclusion

In this work, the evaluation of the effect of temperature on the synthesis of new Pd nanoparticle-enzyme biohybrids has been successfully developed. It was demonstrated how the temperature variation directly affects the formation of the particles and therefore their size, obtaining PdNPs in the size range from 2 to 10 nm, from 4 °C to 50 °C of synthesis temperature, respectively. In addition, the thermal phenomenon was critical in the formation of nanobiohybrids with other enzymes, where the relation of the protein structure and temperature and the influence of the latter have been demonstrated to be critical in the reducing efficiency of the enzyme in the final Pd nanoparticle formation, in the metal species, or even in the final size of the nanoparticles. These new Pd nanobiohybrids showed excellent results in the C–H activation of protected l-tryptophan under mild conditions. In all the cases, they were selective for *C*-2 arylation synthesis. With the hybrid synthesized at 4 °C using 4-methoxybenzenediazonium tetrafluoroborate as the aryl donor, a conversion greater than 99% in methanol at room temperature was obtained in 1 h, exhibiting a TOF value of 64 min^−1^, 2 or 4 times higher than that of the other synthesized hybrids. This catalyst showed very high recyclability, maintaining >95% activity after three cycles of use. Therefore, these results demonstrate the versatility of these nanobiohybrids in the C–H activation reaction in sustainable chemical processes. This methodology opens the door to applications in the synthesis of more complex molecules and successful application in pharmaceutical and medical chemistry.

## Conflicts of interest

There are no conflicts to declare.

## Supplementary Material

NA-005-D2NA00742H-s001
