# Supplementary material for: Temperature-induced formation of Pd nanoparticles in heterogeneous nanobiohybrids: application in C–H activation catalysis
Source: Nanoscale Adv. 2022 Dec 20;5(2):513–21. doi: 10.1039/d2na00742h (PMC9846520; doi:10.1039/d2na00742h)
Supplement: NA-005-D2NA00742H-s001 [file NA-005-D2NA00742H-s001.pdf]

Electronic Supplementary Material (ESI) for Nanoscale Advances.
This journal is © The Royal Society of Chemistry 2022

Electronic Supplementary Information

Temperature-induced formation of Pd nanoparticles in heterogeneous nanobiohybrids: application in C-H activation catalysis

Noelia Losada-Garcia[a, †], A. Sofia Santos[a,b, †], M. Manuel B. Marques[b] and Jose M. Palomo*[a]

[a] Instituto de Catálisis y Petroleoquímica (ICP), CSIC. C/Marie Curie 2. 28049 Madrid (Spain). *E-mail: josempalomo@icp.csic.es

[b] LAQV@REQUIMTE, Department of Chemistry, NOVA School of Science and Techonology. Universidade Nova de Lisboa, 2829-516 Caparica (Portugal).

[†] Both authors contributed equally.

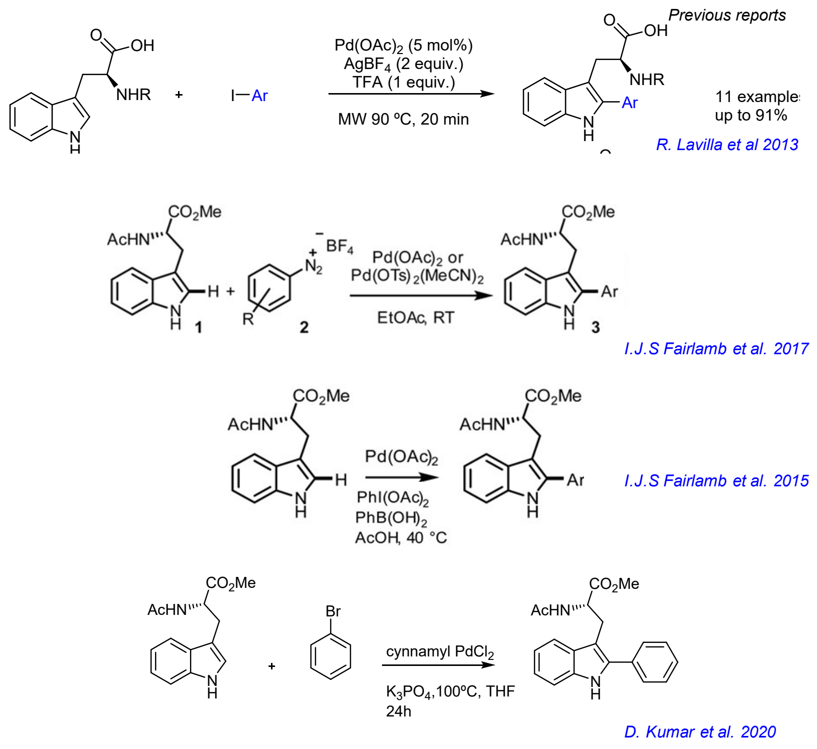

Scheme S1. Selected examples of recent reports of C-H activation of tryptophan derivatives catalysed by Pd molecules.

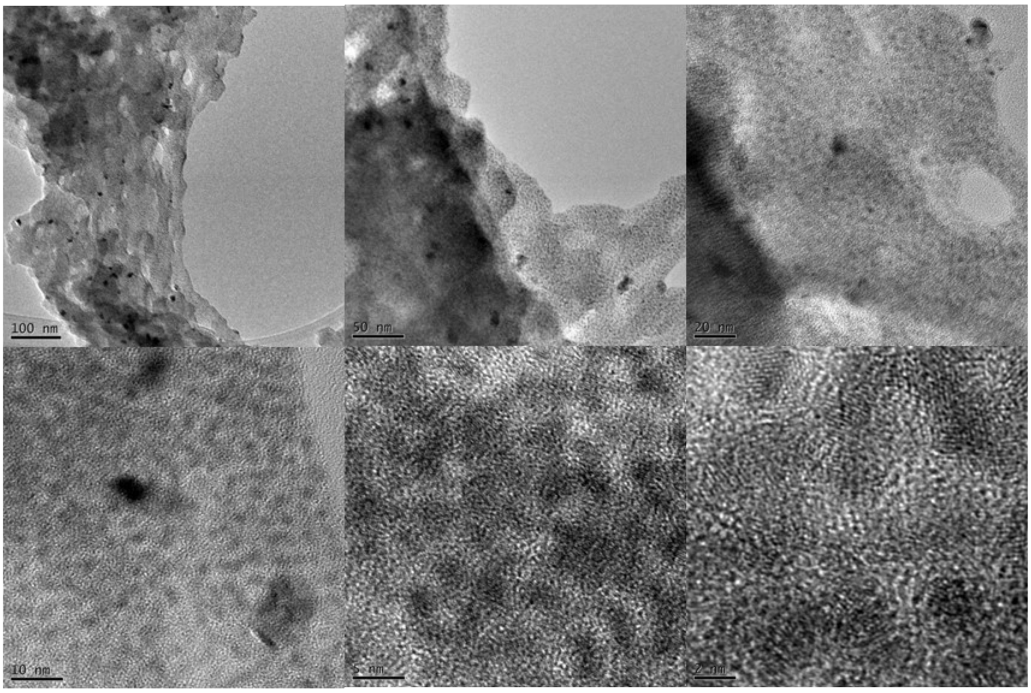

Figure S1. TEM and HR-TEM images of **Pd@CALB4.**

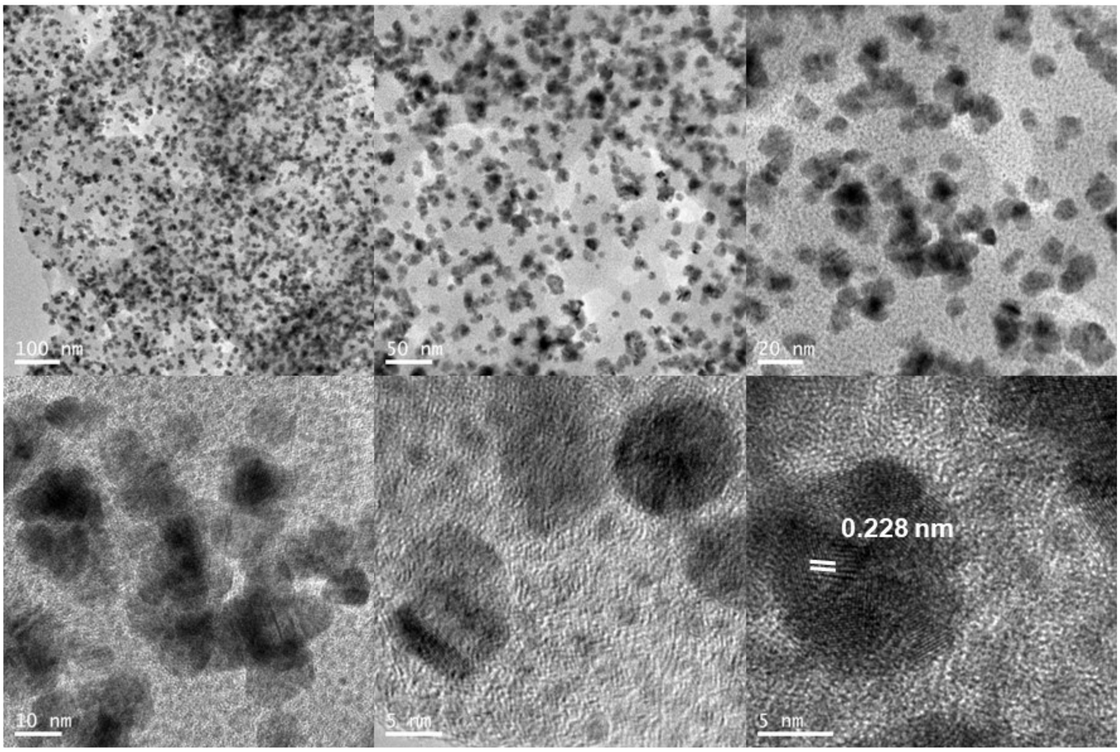

Figure S2. TEM and HR-TEM images of **Pd@CALB50.**

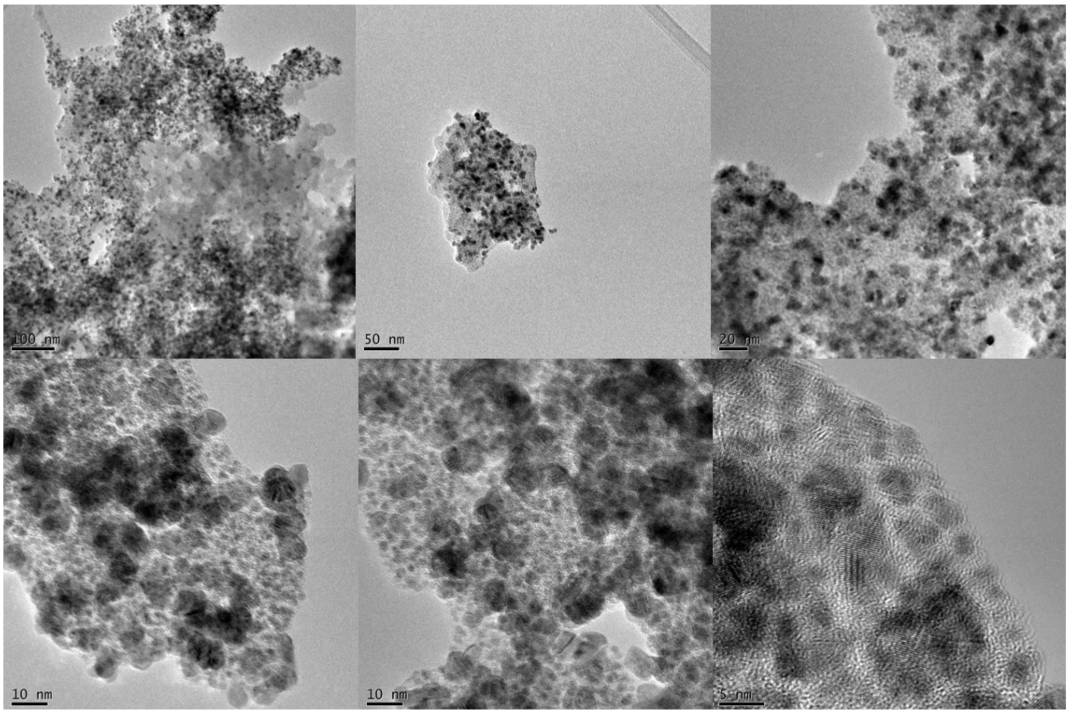

Figure S3. TEM and HR-TEM images of **Pd@CALB60.**

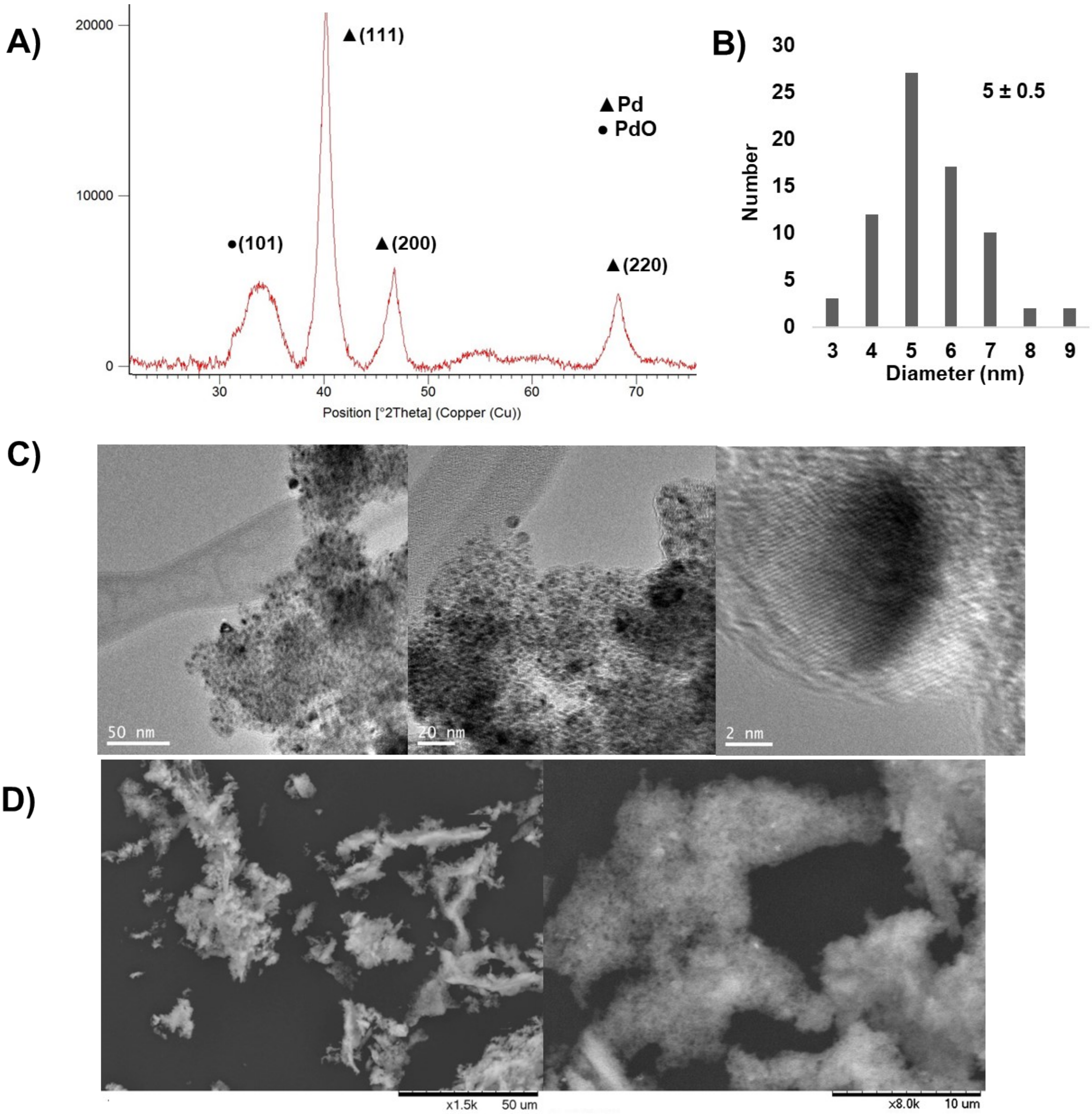

Figure S4. Characterization of **Pd@TLL25. A)** XRD spectrum; **B)** Particle size distribution; **C)** TEM and HR-TEM images; **D)** SEM images.

10	20	30	40	50
MRSSLVLFFV	SAWTALASPI	RREVSQDLFN	QFNLFAQYSA	AAYCGKNNDA
60	70	80	90	100
PAGTNITCTG	NACPEVEKAD	ATFLYSFEDS	GVGDVTGFLA	LDNTNKLIVL
110	120	130	140	150
SFRGSRSIEN	WIGNLNFDLK	EINDICSGCR	GHDGFTSSWR	SVADTLRQKV
160	170	180	190	200
**ED**AVR**EH**P**D**Y	RVVFTG**H**SLG	GALATVAGAD	LRGNGYDIDV	FSYGAPRVGN
210	220	230	240	250
RAFAEFLTVQ	TGGTLYRIT**H**	TNDIVPRLPP	R**E**FGYS**H**SSP	EYWIKSGTLV

PVTRNDIVKI EGIDATGGNN QPNIP**D**IPA**H** LWYFGLIGTC L

Length:291

Mass (Da):31,807

Figure S5. Amino acids sequence of TLL. Asp, Glu and His in bold. Sequence was obtained from https://www.uniprot.org/uniprot/O59952

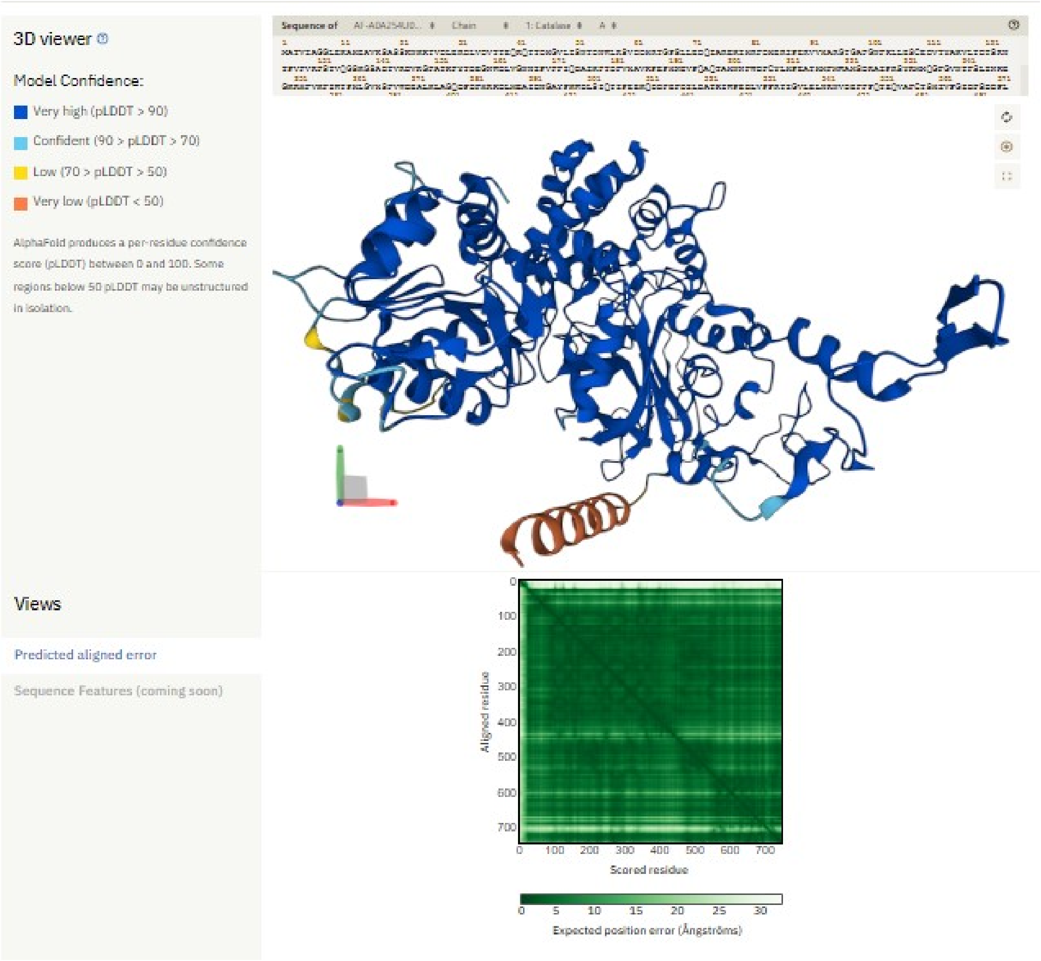

Figure S6. Predicted structure of catalase by AlphaFold, artificial intelligent system developed by DeepMind.

a)

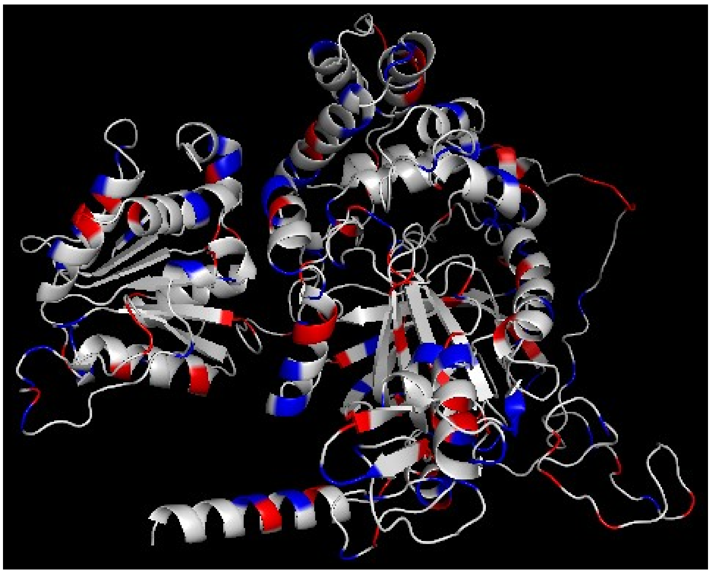

b)

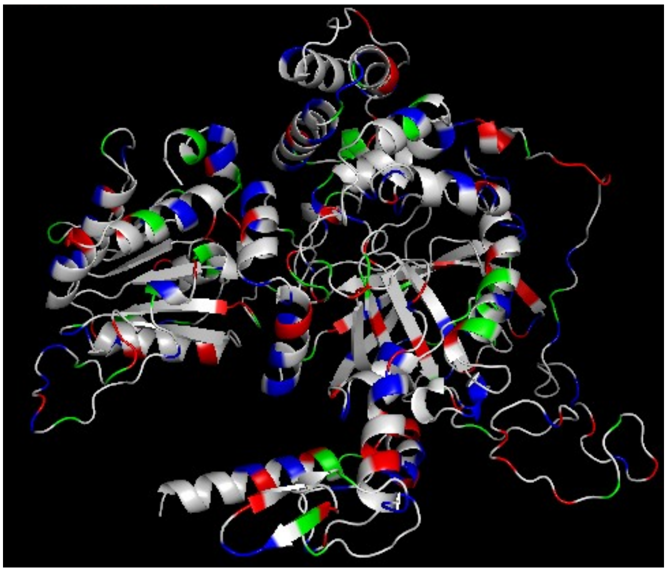

Figure S7. Three-dimensional structure of monomer of tetrameric catalase from *Aspergillus niger*. Structure predicted by AlphaFold and PDB was from AlphaFold. https://alphafold.ebi.ac.uk/entry/A0A254U0S5. Jumper, J *et al*. Highly accurate protein structure prediction with AlphaFold. *Nature* (2021). Image was prepared using Pymol 2.0 software. A) Image structure with Asp and Glu (blue) and Lys and Arg (red). C) Image structure with Asp and Glu (blue), Lys and Arg (red) and Cys, Ser and Tyr (green).

A)

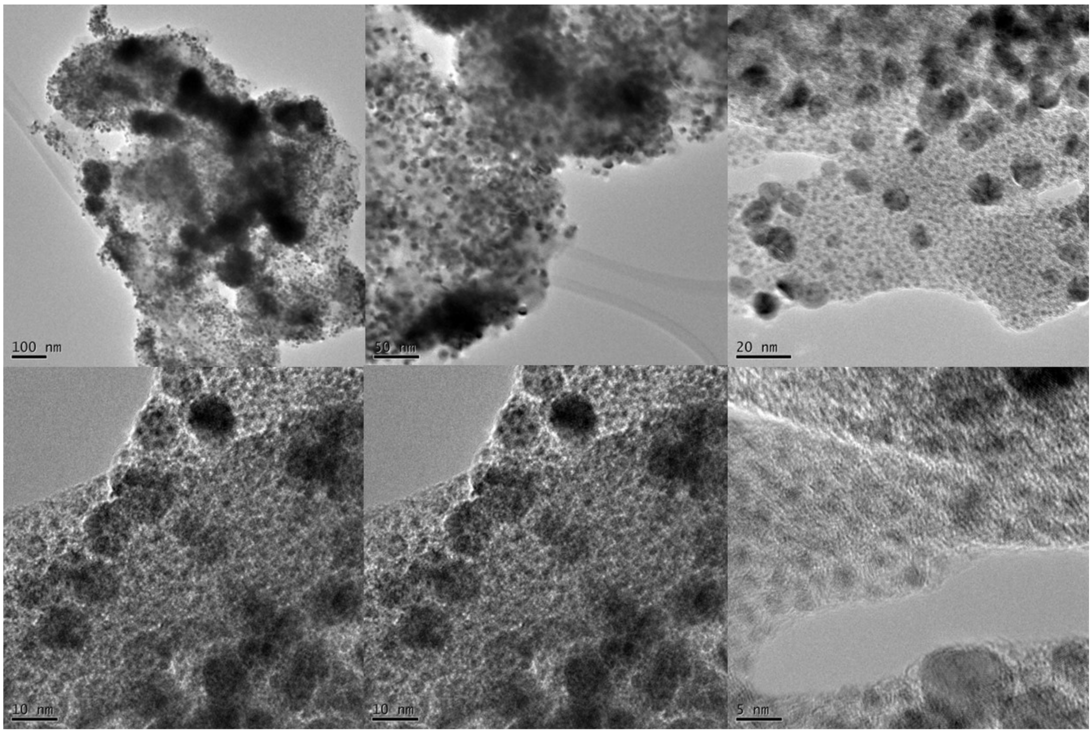

B)

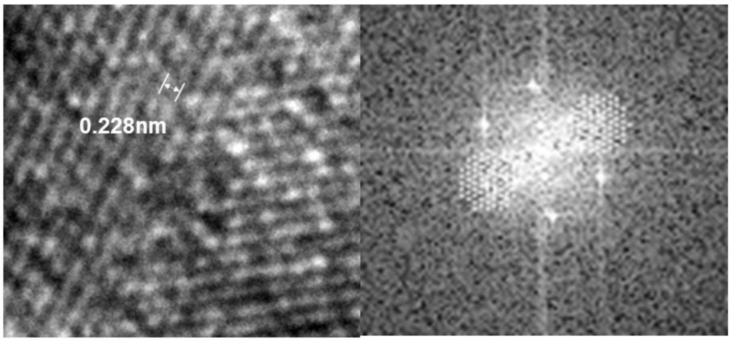

Figure S8. Characterization of **Pd@CAT50. A)** TEM and HR-TEM images; **B)** FFT and IFFT analysis.

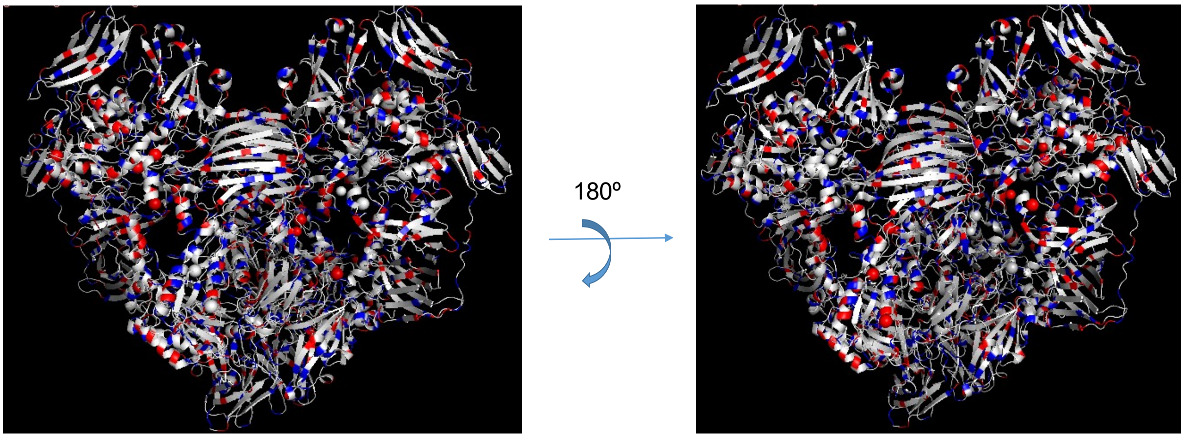

Figure S9. Three-dimensional structure of beta-galactosidase from *Kluyveromyces lactis.* 3OB8 from PDB bank. Structure determined in ref: Pereira-Rodriguez, A., Fernandez-Leiro, R., Gonzalez-Siso, M.I., Cerdan, M.E., Becerra, M., Sanz-Aparicio, J. *Structural basis of specificity in tetrameric Kluyveromyces lactis* beta-galactosidase. J Struct Biol. 2012, 177, 392-401. Asp and Glu (blue), Lys, Arg (red). Mn, Mg (grey balls), Na ions (red balls).

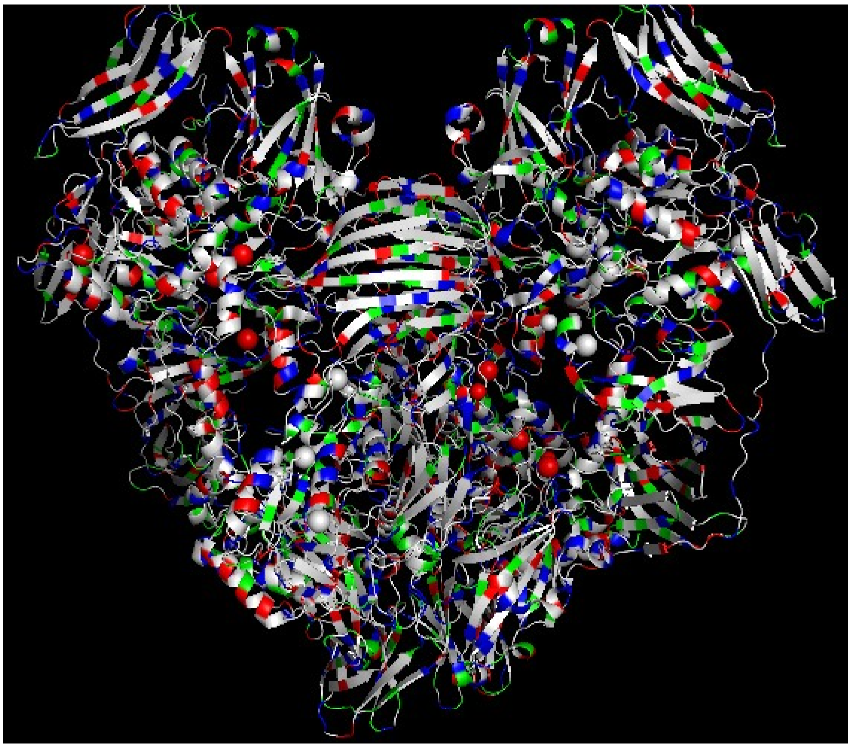

Figure S10. Three-dimensional structure of beta-galactosidase from *Kluyveromyces lactis*. 3OB8 from PDB bank. Structure determined in ref: Pereira-Rodriguez, A., Fernandez-Leiro, R., Gonzalez-Siso, M.I., Cerdan, M.E., Becerra, M., Sanz-Aparicio, J. Structural basis of specificity in tetrameric *Kluyveromyces lactis* beta-galactosidase. J Struct Biol. 2012, 177, 392-401. Asp and Glu (blue), Lys, Arg (red) and Cys, Ser and Tyr (green). Mn, Mg (grey balls), Na ions (red balls).

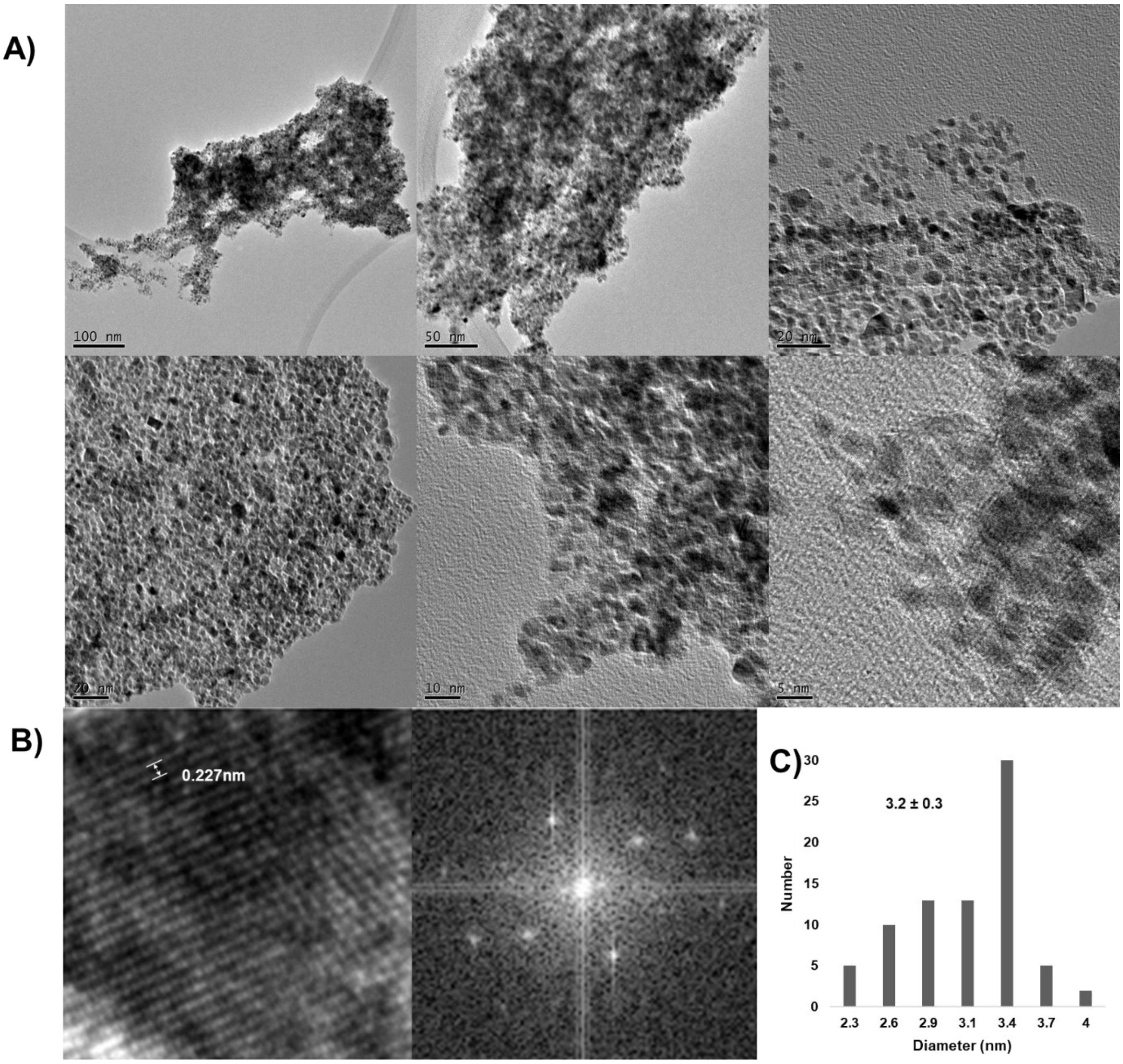

Figure S11. Characterization of **Pd@LAC70. A)** TEM and HR-TEM images; **B)** FFT and IFFT analysis; **C)** Particle size distribution.

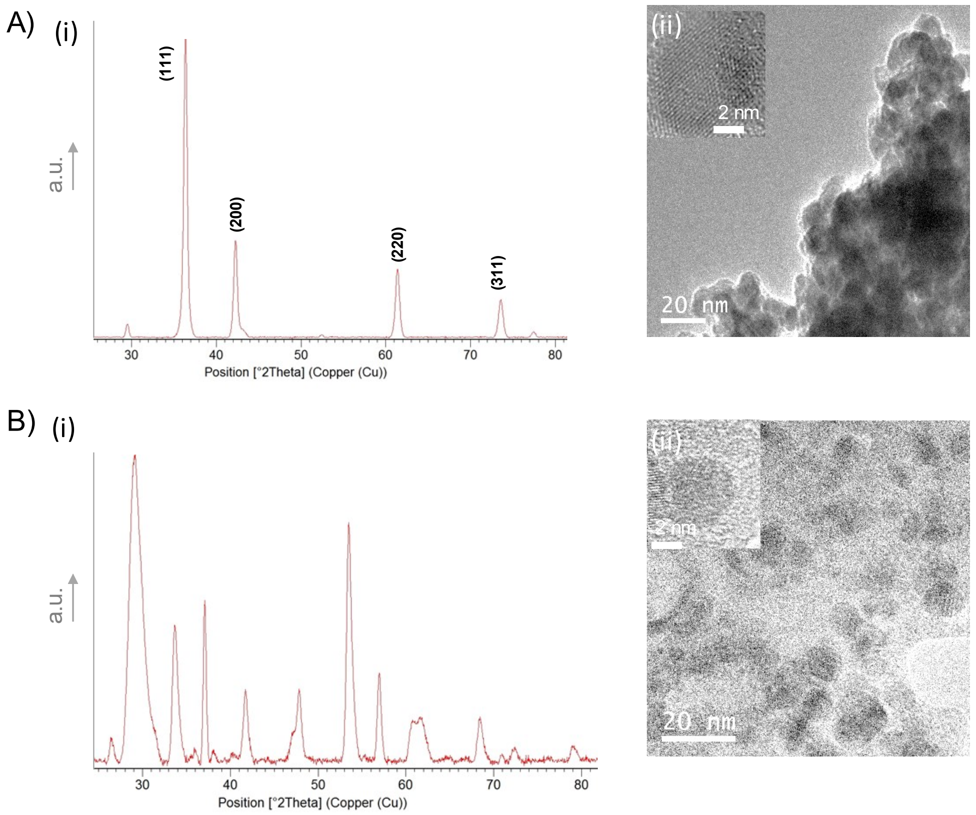

Figure S12. Characterization of Cu hybrids. A) Cu-FOS2. B) Cu-NR. i) XRD analysis, ii) TEM image (inset HRTEM).

Table S1. Determination of Pd% and yield of Pd deposition in the different Pd@enzyme hybrids.

**Pd Hybrids**	**Pd employed (µmol)**	**Pd content (w/w %)[a]**	**Amount of Hybrid (mg)**	**Pd Deposited (µmol)**	**Pd Deposition yield (%)**
**Pd@CALB4**	188	15	23	32.43	17
**Pd@CALB25**	188	26	24	58.65	31
**Pd@CALB50**	188	22	16	33.08	18
**Pd@CALB60**	188	22	23	47.56	25
**Pd@TLL25**	188	35	14	46.06	24
**Pd@CAT50**	188	33	0.6	1.86	1
**Pd@LAC70**	188	64	9.4	56.55	30

[a] Determined by ICP_OES analysis. The measurement was performed of the solid material. 1 mg of the solid powder was treated with 5 mL of HCl (37% v/v) for digestion. Then, it was added with 5 mL of water, centrifuged and the clear solution analyzed by Pd content.
